# TRF1 phosphorylation on T271 modulates telomerase-dependent telomere length maintenance as well as the formation of ALT-associated PML bodies

**DOI:** 10.1038/srep36913

**Published:** 2016-11-14

**Authors:** Angus Ho, Florence R. Wilson, Stephanie L. Peragine, Kajaparan Jeyanthan, Taylor R. H. Mitchell, Xu-Dong Zhu

**Affiliations:** 1 Department of Biology, McMaster University, Hamilton, Ontario L8S 4K1, Canada

## Abstract

TRF1, a component of the shelterin complex, plays a key role in both telomerase-dependent telomere maintenance and alternative lengthening of telomeres, the latter also known as ALT. Characteristics of ALT cells include C-circles and ALT-associated PML bodies, referred to as APBs. The function of TRF1 is tightly regulated by post-translational modification including phosphorylation, however TRF1 phosphorylation sites have yet to be fully characterized. Here we report a novel TRF1 phosphorylation site threonine 271. We show that a nonphosphorylatable mutation of T271A impairs TRF1 binding to telomeric DNA *in vivo* and renders TRF1 defective in inhibiting telomerase-dependent telomere elongation. On the other hand, TRF1 carrying a phosphomimic mutation of T271D is competent in not only binding to telomeric DNA but also inhibiting telomerase-mediated telomere lengthening. These results suggest that TRF1 phosphorylation on T271 negatively regulates telomerase-mediated telomere maintenance. We find that in telomerase-negative ALT cells, TRF1 carrying either a T271A or T271D mutation is able to promote C-circle production but fails to support APB formation. These results suggest that TRF1 phosphorylation on T271 is necessary for APB formation but dispensable for C-circle production. These results further imply that APB formation can be mechanistically separated from C-circle production.

Telomeres, specialized heterochromatic structures found at the ends of linear eukaryotic chromosomes, are vital for cell survival and proliferation. Most human somatic cells encounter an erosion of telomeric DNA every time they replicate their genome, in part due to an inability of DNA polymerases to fill in the gap left from removal of the last RNA primer^1^. Following a limited number of population doublings (PDs), these cells will ultimately enter replicative senescence as a result of the activation of DNA damage response trigged by critially short telomeric DNA^2^. About 85–90% of human cancers avoid replicative senescence and gain unlimited growth potential by activating telomerase^3^. The remaining 10–15% of human cancers do not activate telomerase but instead maintain their telomere length through a homologous recombination (HR)-based mechanism, referred to as *a*lternative *l*engthening of *t*elomeres (ALT)^4^. The ALT pathway can also be induced in tumors upon telomerase extinction^5^. Thus understanding the mechansims underlying both telomerase-dependent telomere length maintenance as well as the ALT pathway will be of importance in the design of therapeutic approaches targeting both telomerase and ALT simultaneously.

ALT cells carry several hallmarks that differentiate them from telomerase-expressing cells^4^. These include PML bodies containing telomeric chromatin, referred to as ALT-associated PML bodies (APBs), as well as a high level of extra-chromosome telomeric DNA in the form of double stranded linear/circular DNA or single-stranded circular DNA such as C-circles^6^.

Mammalian telomeres are coated with a six-subunit protein complex, known as shelterin/telosome, which consists of TRF1, TRF2, TIN2, hRap1, TPP1 and POT1^7^,^8^. The shelterin complex plays a critical role in maintaining telomere length homeostasis as well as protecting telomere ends from being recognized as damaged DNA. TRF1, the first cloned shelterin subunit^9^, binds to duplex telomeric DNA in a sequence-specific manner. In telomerase-expressing cells, TRF1 acts as a negative regulator of telomere length maintenance^10^,^11^. Depletion of TRF1 or overexpression of a dominant-negative allele of TRF1 promotes telomerase-dependent telomere lengthening whereas overexpression of TRF1 induces telomere shortening^10^,^11^,^12^. On the other hand, in ALT cells, TRF1 is implicated as a positive regulator of HR-based telomere maintenance^13^. Depletion of TRF1 impairs the formation of APBs and C-circle production^13^,^14^. TRF1 undergoes extensive phosphorylation, which has been implicated in the regulation of TRF1 binding to telomeric DNA as well as its stability in telomerease-expressing cells^15^. Recent work also suggests that TRF1 phosphorylation modulates APB formation and C-circle production in ALT cells^14^.

In this report, we demonstrate that TRF1 is phosphorylated on threonine 271 *in vivo*. We show that in telomerase-positive cells, overexpression of TRF1 carrying a nonphosphorylatable mutation of T271A fails to inhibit telomere elongation whereas overexpression of TRF1 carrying a phosphomimic mutation of T271D is able to suppress telomere lengthening. We further demonstrate that TRF1 carrying a T271A mutation is defective in binding to telomeric DNA *in vivo*, which may at least in part account for the inability of TRF1-T271A to suppress telomerase-mediated telomere elongation. We find that in ALT cells, TRF1 carrying either a T271A or T271D mutation is able to support the production of C-circles, indicating that T271 is not involved in regulating the level of C-circles. On the other hand, TRF1 carrying a T271A mutation is deficient in supporting APB formation, indicative of the role of T271 phosphorylation in APB formation, suggesting that the role of TRF1 in supporting APB formation can be separated from its role in promoting C-circle production in ALT cells. Taken together, the work presented here suggests that TRF1 phosphorylation on T271 plays an important role in modulating its activity in both telomerease-expressing and ALT cells.

## Results

### TRF1 is phosphorylated on T271 *in vivo*

**Figure 1:**
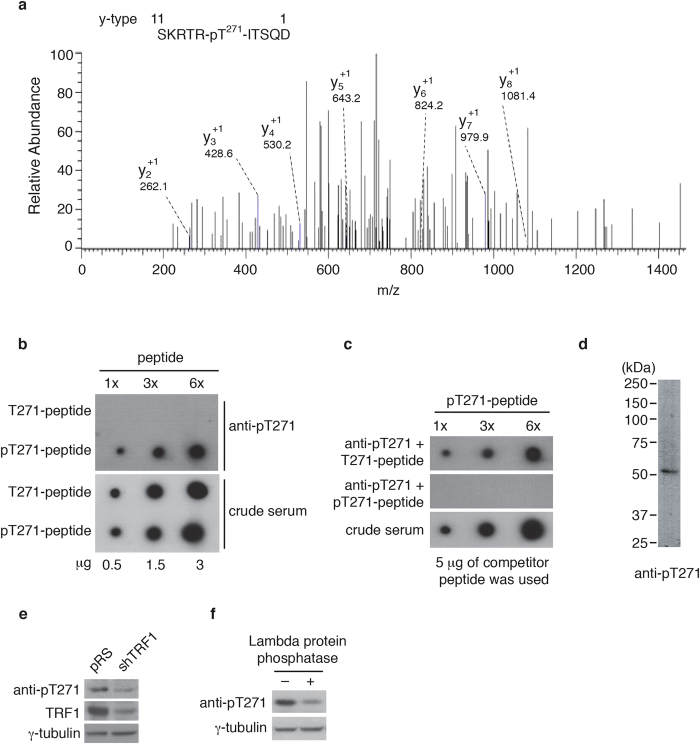
T271 of TRF1 is phosphorylated *in vivo*. (**a**) LC/MS/MS analysis was performed on Flag-tagged TRF1 immunoprecipitated from HT1080 cells. The spectrum of the peptide identified to contain phosphorylated threonine at position 271 is shown with the relative abundance plotted against the monoisotopic mass (m/z). The m/z peaks from the y-type ions are indicated. Mass spectrometry analysis of Flag-tagged TRF1 was done through service provided by WEMB Biochem. Inc. Toronto, Canada. (**b**) Affinity-purified anti-pT271 antibody specifically recognizes TRF1 peptide containing phosphorylated T271 (pT271-peptide). An increasing amount of peptide either carrying unmodified T271 (pT271-peptide) or phosphorylated T271 (pT271-peptide) was spotted on a nitrocellulose membrane, followed by immunoblotting with affinity-purified anti-pT271 antibody or crude serum. The amount of peptide spotted from left to right is 0.5 μg, 1.5 μg and 3 μg. (**c**) Peptide competition assays. Affinity-purified anti-pT371 antibody was incubated with 5 μg of either unmodified (T271-peptide) or phosphorylated (pT271-peptide) prior to immunoblotting. The blot with crude serum was used to indicate the presence of pT271-peptide on the membrane. The amount of pT271-peptide spotted from left to right is the same as described in (**b**). (**d**) Western analysis of HeLaII whole cell lysate with anti-pT271 antibody. (**e**) Western analysis of HeLII cells depleted for endogenous TRF1. Immunoblotting was performed with anti-pT271 and anti-TRF1 antibody. The γ-tubulin blot was used as a loading control in this experiment and all following westerns shown in this article. (**f**) Western analysis. HeLa nuclear extract (75 μg) was treated in the presence or absence of Lambda protein phosphatase (800 units, NEB). Immunoblotting was performed with anti-pT271 and anti-γ-tubulin antibodies.

Mass spectrometry analysis of immunoprecipitated Flag-TRF1 showed that threonine 271 of TRF1 was a candidate phosphorylation site (Fig. 1a). To further investigate TRF1 phosphorylation on T271 *in vivo*, we raised an antibody specifically against a TRF1 peptide containing phosphorylated T271 (Fig. 1b,c). Western analysis revealed that the anti-pT271 antibody predominantly recognized a protein band with an apparent molecular weight indistinguishable from that of endogenous TRF1 (Fig. 1d). Depletion of TRF1 resulted in a loss of TRF1 recognized by anti-pT271 antibody (Fig. 1e). Furthermore, the anti-pT271 signal was sensitive to the treatment with Lambda protein phosphatase (Fig. 1f). Taken together, these results reveal that TRF1 is phosphorylated on T271 *in vivo*.

### TRF1 phosphorylation on T271 is needed to suppress telomerase-dependent telomere lengtheningn

**Figure 2:**
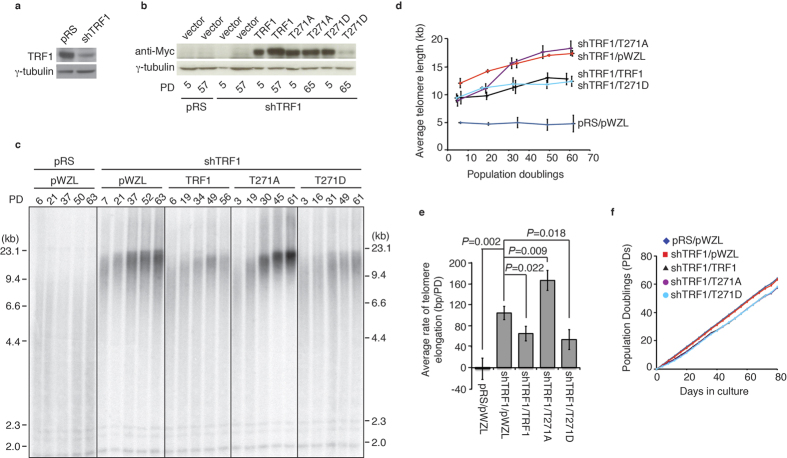
TRF1 carrying a nonphosphorylatable mutation of T271A is defective in inhibiting telomerase-dependent telomere lengthening. (**a**) Western analysis of HeLaII cells expressing either the vector pRS alone or shRNA against TRF1 (shTRF1). Immunoblotting was performed with anti-TRF1 and anti-γ-tubulin antibodies. (**b**) Western analysis of pRS- and shTRF1-expressing HeLaII cells expressing the vector pWZL-NMyc vector alone or various TRF1 alleles as indicated above the lanes. Immunoblotting was performed with anti-Myc and anti-γ-tubulin antibodies. (**c**) Genomic blots of telomeric restriction fragments from TRF1-depleted HeLaII cells (shTRF1) expressing either the vector pWZL alone or various TRF1 alleles as indicated above the lanes. HeLaII cells expressing both pRS and pWZL were used as a control. PD are indicated above the lanes whereas DNA molecular weight markers are shown on both left and right of the blots. About 3 μg of *Rsa*I/*Hinf*I-digested genomic DNA from each sample was used for gel electrophoresis. (**d**) Average telomere length of indicated cell lines was ploted against PD. Standard deviations from three independent experiments are indicated. (**e**) Quantification of average rates of telomere elongation of indicated cell lines. Standard deviations from three independent experiments are indicated. (**f**) Growth curve of HeLaII cells expressing various constructs as indicated from one of the three independent experiments. The number of PDs was plotted against days in culture.

It has been well established that TRF1 acts as a negative mediator of telomerase-dependent telomere lengthening^10^,^11^,^12^. To investigate the role of TRF1 phosphorylation on T271 in telomere length maintenance, we asked if either a nonphosphorylatable mutation of T271A or a phosphomimic mutation of T271D might affect telomerase-dependent telomere lengthening. To address this question, we generated HeLaII cells that stably expressed either the vector alone or shRNA against TRF1 (shTRF1) (Fig. 2a). TRF1-depleted HeLaII cells were then infected with retrovirus expressing the vector pWZL alone, Myc-tagged wild type TRF1, Myc-tagged TRF1 carrying a nonphosphorylatable mutation of T271A or Myc-tagged TRF1 carrying a phosphomimic mutation of T271D, giving rise to four stable cell lines (shTRF1/pWZL, shTRF1/TRF1, shTRF1/T271A, shTRF1/T271D) (Fig. 2b). As a control, we also generated a HeLaII cell line stably expressing two vectors pRetroSuper (pRS) and pWZL. We chose to use TRF1-depleted cells for analysis of TRF1 mutants to minimize the interference from endogenous TRF1^12^,^16^. Myc-tagged wild type TRF1, Myc-tagged TRF1-T271A and Myc-tagged TRF1-T271D contained silent mutations that rendered them resistant to shTRF1.

These stable cell lines, which contained pools of cells and are not single cell clones, were cultured continuously for over 56 population doublings (PDs). Depletion of TRF1 resulted in telomere elongation (Fig. 2c,d), in agreement with previous findings^12^. The average rate of telomere elongation for TRF1-depleted cells expressing the vector alone was about 104 bp per PD over a period of 63 PDs (Fig. 2e). Introduction of shTRF1-resistant wild type TRF1 suppressed telomere elongation in TRF1-depleted HeLaII cells (Fig. 2c,d) and reduced the average rate of telomere elongation in shTRF1-depleted cells from 104 bp per PD to about 64 bp per PD (Fig. 2e), in agreement with previous finding that TRF1 acts as a negative mediator of telomerase-dependent telomere lengthening^10^,^12^. On the other hand, introduction of shTRF1-resistant TRF1-T271A failed to suppress telomere elongation in TRF1-depleted cells (Fig. 2c,d). We found that the average rate of telomere elongation in shTRF1-depleted cells complemented with TRF1-T271A was about 166 bp/PD over a period of 61 PDs (Fig. 2e), significantly higher than that in shTRF1-depleted cells complemented with the vector alone (about 104 bp/PD) (Fig. 2e). Introduction of shTRF1-resistant TRF1-T271D was able to suppress telomere elongation in TRF1-depleted HeLaII cells in a manner similar to wild type TRF1 (Fig. 2c,e). The average rate of telomere elongation in TRF1-depleted cells complemented with TRF1-T271D was about 53 bp/PD over a period of 61 PDs (Fig. 2e). The inability of TRF1-T271A to reverse shTRF1-mediated telomere lengthening was unlikely due to the lack of protein expression (Fig. 2b). TRF1-T271A-expressing cells grew at a rate indistinguishable from wild type TRF1- or TRF1-T271D-expressing cells (Fig. 2f), arguing against the possibility that the difference in cell proliferation may account for the inability of TRF1-T271A to inhibit telomere lengthening. Taken together, these results suggest that TRF1 phosphorylation on T271 is needed for TRF1 to negatively regulate telomerase-dependent telomere lengthening.

### TRF1 phosphorylation on T271 facilitates its association with telomeric DNA *in vivo*

**Figure 3:**
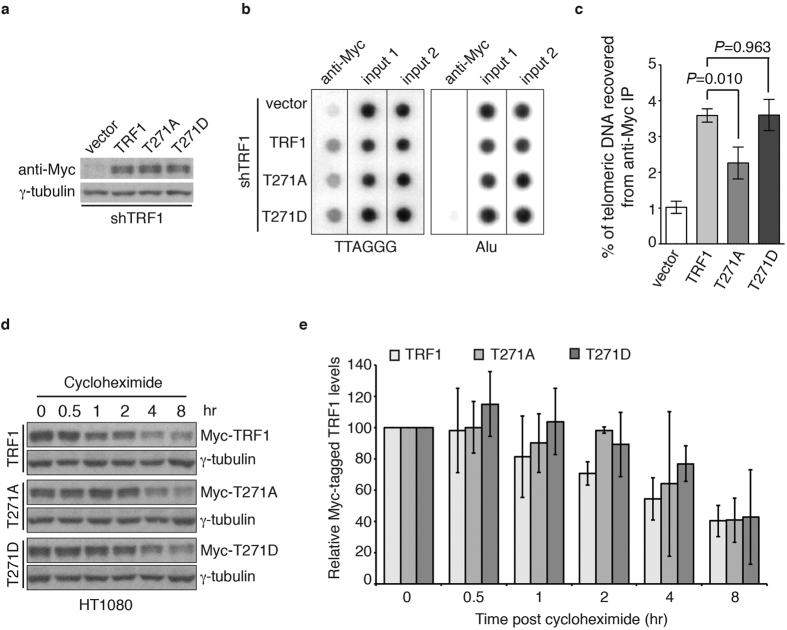
TRF1 carrying a nonphosphorylatable mutation of T271A is defective in binding to telomeric DNA *in vivo*. (**a**) Western analysis of TRF1-depleted HeLaII cells expressing the pWZL vector alone or varous TRF1 alleles as indicated. Immunoblotting was performed with anti-Myc and anti-γ-tubulin antibodies. (**b**) Dot blots of anti-Myc ChIPs from TRF1-depleted HeLaII cells expressing various constructs as indicated on the left. (**c**) Quantification of anti-Myc ChIPs from (**b**). Standard deviations from three independent experiments are indicated. (**d**) Cycloheximide chase experiments. HT1080 cells expressing Myc-TRF1, Myc-TRF1-T271A or Myc-TRF1-T271D were treated with 100 μg/ml cycloheximide for the indicated times, followed by immunoblotting of the lysates with anti-Myc and anti-γ-tubulin antibodies. (**e**) Quantification of Myc-tagged wild type, Myc-tagged TRF1-T271 and Myc-tagged TRF1-T271D from (**d**). The signals from the western blots were quantified with densitometry. The level of Myc-tagged wild type TRF1 and various mutant TRF1 proteins is represented in arbitrary units after their signals were first normalized relative to those of γ-tubulin and then normalized relative to Myc-tagged wild type TRF1. Standard deviations from three independent experiments are indicated.

It has been well documented that TRF1 binding to telomeric DNA negatively regulates telomerase-dependent telomere elongation^10^,^11^,^12^. To investigate if T271 phosphorylation might modulate TRF1 binding to telomeric DNA *in vivo*, we performed analysis of chromatin immunoprecipitation (ChIP) using an anti-Myc antibody and lysates isolated from early passages of TRF1-depleted HeLaII cells stably expressing the vector alone, Myc-tagged wild type TRF1, Myc-tagged TRF1-T271A and Myc-tagged TRF1-T271D. Both Myc-tagged TRF1-T271A and Myc-tagged TRF1-T271D were expressed at a comparable level to Myc-tagged wild type TRF1 (Fig. 3a). Analysis of ChIP with an anti-Myc antibody revealed that while a phosphomimic mutation of T271D did not affect TRF1 association with telomeric DNA, a nonphosphorylatable mutation of T271A significantly impaired TRF1 interaction with telomeric DNA *in vivo* (Fig. 3b,c). These results suggest TRF1 phosphorylation on T271 is needed for its binding to telomeric DNA *in vivo*.

Phosphorylation has been implicated in regulating TRF1 stability^15^, which could in turn affect TRF1 interaction with telomeric DNA. To investigate if T271 phosphorylation might affect TRF1 stability, we performed cycloheximide chase experiments in HT1080 cells stably expressing the vector alone, Myc-tagged wild type TRF1, Myc-tagged TRF1-T271A and Myc-tagged TRF1-T271D. We found that Myc-tagged TRF1-T271A was as stable as Myc-tagged wild type TRF1 and Myc-tagged TRF1-T271D (Fig. 3d,e), indicating that TRF1 phosphorylation on T271 is not involved in the regulation of TRF1 stability. Taken together, these results suggest that the observed reduced ability of Myc-tagged TRF1-T271A to bind telomeric DNA *in vivo* is unlikely due to a defect in its stability. These results further imply that the defect in the ability of Myc-tagged TRF1-T271A to bind telomeric DNA may at least in part account for our earlier observation that Myc-tagged TRF1-T271A fails to suppress telomerase-dependent telomere lengthening in TRF1-depleted HeLaII cells.

### TRF1 phosphorylation on T271 is dispensable for C-circle production.

**Figure 4:**
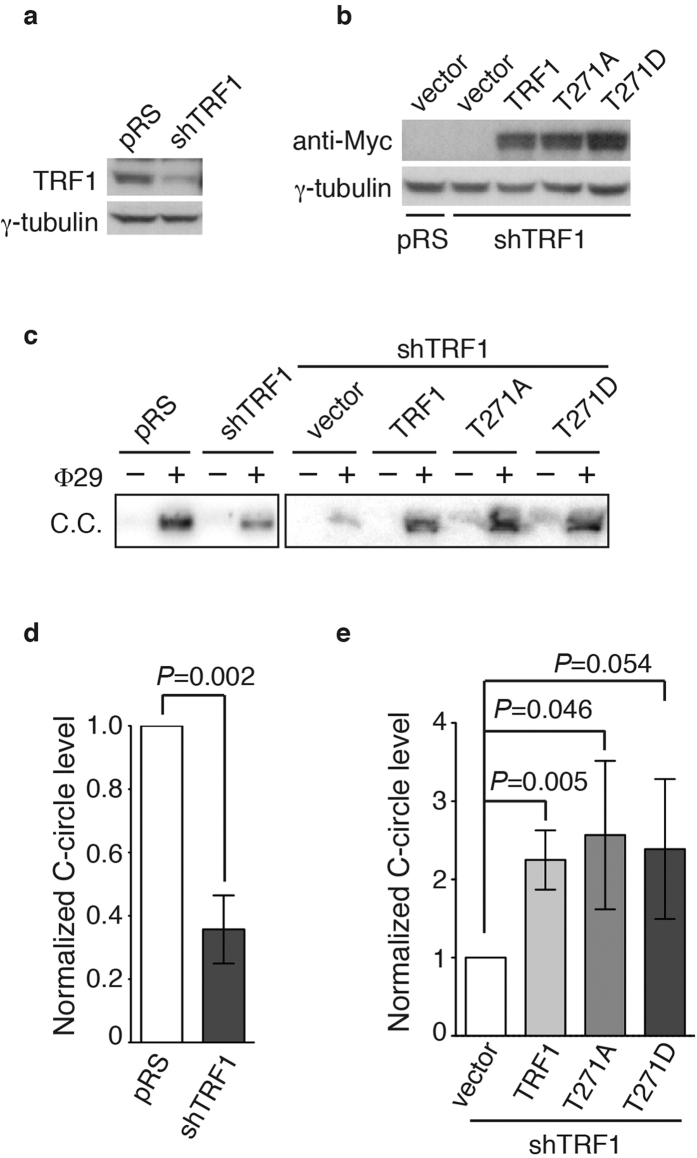
TRF1 phosphorylation on T271 is dispensable for C-circle production in ALT cells. (**a**) Western analysis of GM847 cells stably expressing the vector pRS alone or shRNA against TRF1 (shTRF1). Immunoblotting was performed with anti-TRF1 and anti-γ-tubulin antibodies. (**b**) Western analysis of pRS- and shTRF1-expressing GM847 cells expressing the vector alone (pWZL) or various TRF1 alleles as indicated above the lanes. Immunoblotting was performed with anti-Myc and anti-γ-tubulin antibodies. (**c**) Analysis of C-circle formation. C.C. stands for C-circles. (**d**,**e**) Quantification of the level of C-circles from (**c**). The C-circle signals were quantified with ImageQuant. The level of C-circles is represented in arbitrary units. In (**d**) the signal in the shTRF1 lane was normalized relative to that in the pRS lane whereas in (**e**) the signals in lanes representing Myc-tagged wild type TRF1 and various mutant TRF1 proteins were normalized relative to that in the vector alone lane. Standard deviations from three independent experiments are indicated.

In addition to its role in negatively regulating telomerase-dependent telomere maintenance, TRF1 has been implicated in promoting C-circle production and APB formation^14^, characteristic features associated with alternative lengthening of telomeres. To investigate if TRF1 phosphorylation on T271 might regulate C-circle production, we depleted endogenous TRF1 in GM847 cells (Fig. 4a) and then complemented TRF1-depleted GM847 cells with Myc-tagged shTRF1-resistant wild type TRF1 or TRF1 carrying an amino acid substitution of T271A or T271D. The expression of Myc-tagged TRF1 mutant alleles was comparable to that of Myc-tagged wild type TRF1 (Fig. 4b). Analysis of C-circle assays revealed that depletion of TRF1 resulted in a significant loss in the level of C-circles in GM847 cells (Fig. 4c,d), in agreement with previous finding^14^. Like Myc-tagged wild type TRF1, both Myc-tagged TRF1-T271A and Myc-tagged TRF1-T271D were able to rescue the level of C-circles in TRF1-depleted cells (Fig. 4c,e). These results suggest that T271 of TRF1 is not involved in regulating C-circle production.

### TRF1 phosphorylation on T271 is needed for APB formation

**Figure 5:**
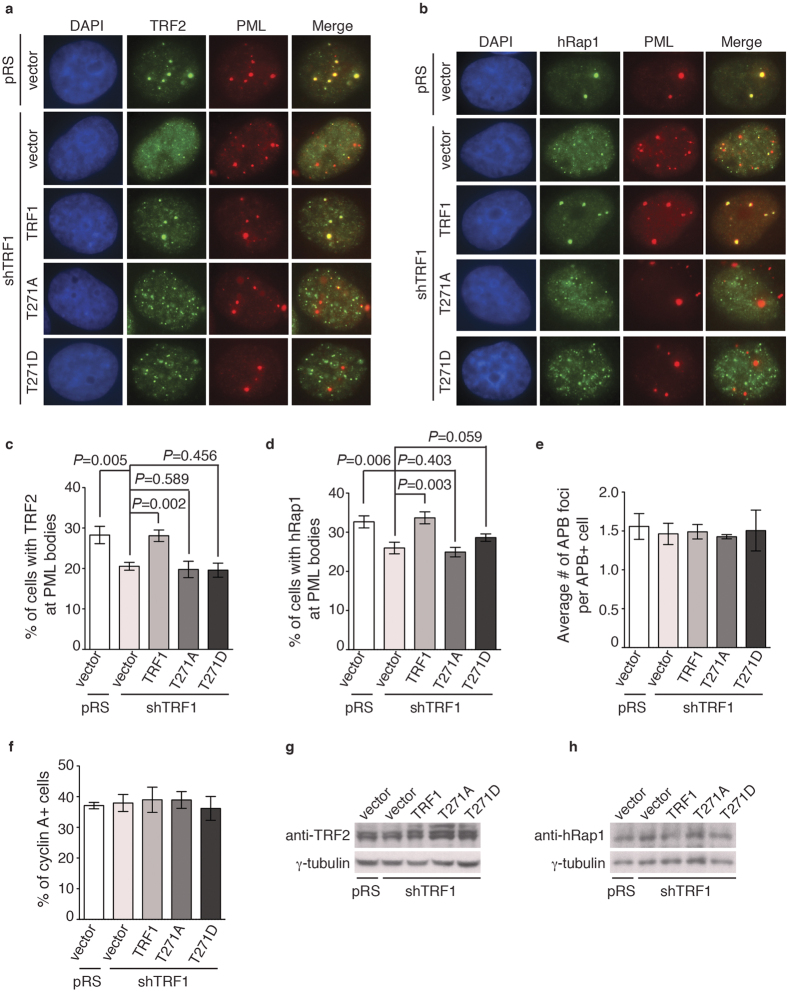
TRF1 phosphorylation on T271 is needed to support APB formation in ALT cells. (**a**) Dual Indirect immunofluorescence with anti-TRF2 and anti-PML antibodies on GM847 cells expressing various constructs as indicated. Cell nuclei are stained with DAPI in blue in this and subsequent figures. (**b**) Dual Indirect immunofluorescence with anti-hRap1 and anti-PML antibodies on GM847 cells expressing various constructs as indicated. (**c**) Quantification of the percentage of cells with TRF2 at APBs from (**a**). A total of 1000 cells from each independent experiments were scored for each cell line in blind. Standard deviations from three independent experiments are indicated. (**d**) Quantification of the percentage of cells with hRap1 at APBs from (**b**). A total of 500 cells from each independent experiments were scored for each cell line in blind. Standard deviations from three independent experiments are indicated. (**e**) Quantification of the average number of APB foci per APB+ cell. A total of at least 250 cells from captured images from three independent experiments were scored in blind. Standard deviations from three independent experiments are indicated. (**f**) Quantification of the percentage of GM847 cells expressing various constructs as indicated staining positive for cyclin A. Scoring was done as described in (**c**). Standard deviations from three independent experiments are indicated. (**g**) Western analysis of GM847 cells expressing various constructs as indicated. Immunoblotting was performed with anti-TRF2 and anti-γ-tubulin antibodies. (**h**) Western analysis of GM847 cells expressing various constructs as indicated. Immunoblotting was performed with anti-hRap1 and anti-γ-tubulin antibodies.

APBs, hallmarks of ALT cells, have originally been described as large PML nuclear bodies containing telomere DNA and shelterin proteins^17^. To investigate if TRF1 phosphorylation on T271 might be involved in the regulation of APB formation, we examined the colocalization of TRF2 and hRap1, components of the shelterin complex, with large PML bodies in TRF1-depleted GM847 cells stably expressing the vector alone, Myc-tagged wild type TRF1 or Myc-tagged TRF1 mutant alleles. Analysis of indirect immunofluorescence revealed that depletion of TRF1 resulted in a significant reduction in the percentage of GM847 cells exhibiting colocalization of large PML bodies with TRF2 and hRap1 (Fig. 5a–d), in agreement with previous finding that TRF1 is required for the formation of APBs^13^,^14^. We observed that among cells scored positive for APBs, the average number of APB foci per cell was about 1.5 (Fig. 5e), which was not significantly affected by depletion of TRF1 or introduction of various TRF1 alleles (Fig. 5e). Introduction of Myc-tagged wild type TRF1 rescued the percentage of TRF1-depleted GM847 cells exhibiting colocalization of large PML bodies with TRF2 and hRap1 (Fig. 5a–d) whereas introduction of Myc-tagged TRF1 carrying a T271A mutation failed to do so (Fig. 5a–d). Overexpression of Myc-tagged TRF1-T271A did not result in any significant change in the percentage of GM847 cells in S and G2 phases (Fig. 5f), arguing against the possibility that the observed inability of Myc-tagged TRF1-T271A to rescue APB formation was due to a change in the cell cycle profile. Furthermore, ovexpression of Myc-tagged TRF1-T271A had little impact on the level of endogenous TRF2 and hRap1 in GM847 cells (Fig. 5g,h). Taken together, these results suggest that T271 phosphorylation is needed to support APB formation. We observed that overexpression of Myc-tagged TRF1 carrying a T271D mutation also failed to rescue the percentage of TRF1-depleted GM847 cells exhibiting the association of TRF2 and hRap1 with large PML bodies (Fig. 5a–d), suggesting that Myc-tagged TRF1-T271D does not mimic T271 phosphorylation in supporting APB formation.

## Discussion

TRF1, a component of the shelterin complex, plays a crucial role in telomere maintenance in both telomerase-positive and telomerase-negative ALT cancer cells. The activity of TRF1 is tightly regulated by post-translational modifications including phosphorylation^15^. Here we report a novel TRF1 phosphorylation site threonine 271 and demonstrate that TRF1 phosphorylation on T271 regulates TRF1 activity not only in telomerase-dependent telomere lengthening but also in the formation of APBs, hallmarks of telomerase-negative ALT cells.

We have shown that introduction of shTRF1-resistant wild type TRF1 suppressed telomere elongation in TRF1-depleted HeLaII cells, although it did not completely block telomere lengthening. The latter may be in part due to the fact that telomeres in shTRF1-depleted cells continued to elongate during the period of 63 PDs. We have observed a difference in the starting telomere lengths among the cell lines derived from the same parental shTRF1-depleted HeLaII cell line. A difference in the starting telomere lengths among cell lines derived from a common parental cell line has also been observed in other studies^12^,^18^,^19^. This phenomenon might be in part due to the nature of telomere length heterogeneity of human cells.

We have observed that introduction of TRF1-T271A leads to a further increase in the average rate of telomere elongation in TRF1-depleted cells. Although how TRF1-T271A might promote a further increase in the rate of telomere elongation is unknown, it is possible that the T271A mutation might act as a dominant negative allele in preventing residual TRF1 from suppressing telomerase-dependent telomere elongation in TRF1-depleted cells.

Phosphorylation has been implicated in modulating TRF1 localization within the nucleus. It has been reported that phosphorylation on S367 targets TRF1 to nuclear proteasome centers in S and G2 phases to promote telomerase-dependent telomere lengthening^12^ whereas phosphorylation on T371 in S and G2 phases shields TRF1 from telomere chromatin^16^. We did not observe cell cycle-dependent regulation of TRF1 phosphorylation on T271 through western analysis (T.R.H.M and X.-D.Z., unpublished data). Although we have raised an antibody against a TRF1 peptide carrying phosphorylated T271, this antibody unfortunately works very poorly in fixed cells, making it unusable for analysis of TRF1 nuclear localization through indirect immunofluorescence. Future studies will be needed to investigate if T271 phosphorylation might regulate TRF1 nuclear localization.

Bioinformatics analysis fails to reveal a clear candidate kinase responsible for T271 phosphorylation. Among numerous potential hits, T271 (RTRT^217^) is found to match the consensus phosphorylation site (RXXS/T) of Chk1 and a related kinase Chk2, which has been reported to phosphorylate sheltein protein TRF2^20^. While Chk1 and Chk2 can phosphorylate T271 *in vitro* (K. Jeyanthan and X.-D.Zhu, unpublished data), depletion or inhibition of Chk1 or a related kinase Chk2 fails to affect T271 phosphorylation *in vivo* (J.R. Walker and X.-D. Zhu, unpublished data), arguing against the possibility that Chk1 and Chk2 are the kinases responsible for phosphorylating T271 *in vivo*. Therefore further investigation will be needed to identify the kinase(s) responsible for T271 phosphorylation *in vivo*.

TRF1 contains a N-terminal acid domain, a central TRFH dimerization domain, a linker region and a C-terminal Myb-like DNA binding domain^10^. TRF1 is known to make direct contacts with telomeric DNA through its Myb-like DNA binding domain^21^,^22^ whereas the linker region, which lies between the TRFH domain and the Myb-like DNA binding domain, has been implicated in modulating TRF1 interaction with telomeric DNA. It has been reported that TRF1 phosphorylation on S367 or T371, both of which are located in the linker region, negatively regulates its binding to telomeric DNA^12^,^16^. Our finding that TRF1 phosphorylation on T271, which also lies in the linker region, does not affect its stability but is necessary for its binding to telomeric DNA, suggests that the linker region can also positively regulate TRF1 interaction with telomeric DNA. We have shown that TRF1 phosphorylation on T271 is necessary to inhibit telomerase-dependent telomere lengthening. Interestingly, it has been suggested that Akt phosphorylates T273, which is adjacent to T271, and induces telomere shortening^23^. Whether TRF1 phosphorylation on both T271 and T273 might act synergistically to control telomerase-dependent telomere lengthening requires future studies.

TRF1 has been implicated in C-circle production and APB formation, hallmarks of ALT cells^14^. Recently it has been reported that TRF1 phosphorylation on T371 is required to support both APB formation and C-circle production^14^. We have observed that either a nonphosphorylatable mutation of T271A or a phosphomimic mutation of T271D does not affect TRF1 phosphorylation on T371 (A.H. and X.-D.Z., unpublished data). Our finding that T271 phosphorylation is dispensable for C-circle production but is necessary for APB formation suggests that APB formation may be mechanistically separated from C-circle production.

We have shown that TRF1-T271D acts as a phosphomimic mutant to support its binding to telomeric DNA. In agreement with the notion that TRF1 binding to telomeric DNA is sufficient to block telomerase-dependent telomere lengthening^10^,^11^,^12^, TRF1-T271D is able to inhibit telomerase-mediated telomere length maintenance. On the other hand, TRF1-T271D fails to behave like a phosphomimic mutant to support APB formation in telomerase-negative ALT cells. It has been reported that APB formation requires not only telomere-bound TRF1 but also telomere-free phosphorylated (pT371)TRF1 that interacts with damged DNA but does not bind telomeric DNA sequence per se^14^. We have observed that the T271D mutation does not affect TRF1 phosphorylation on T371 (A.H and X.-D.Z., unpublished data). TRF1 is also reported to be SUMOylated and SUMOylation has been suggested to promote APB formation^24^. Whether T271 might be involved in the regulation of TRF1 SUMOylation requires future investigation.

## Methods

### DNA constructs and antibodies

The DNA construct expressing shRNA against TRF1 (pRS-shTRF1) has been previously described^16^. The QuickChange site-directed mutagenesis kit (Stratagene) was used to create TRF1 mutations of T271A and T271D as well as silent mutations resistant to shTRF1. Wild type TRF1 and TRF1 mutants (T271A and T271D) carrying silent mutations resistant to shTRF1 were then subcloned into the retroviral vector pWZL-N-Myc as described^12^.

Rabbit polyclonal anti-pT271 antibody was developed by Biosynthesis Inc. against a TRF1 peptide containing phosphorylated threonine 271 (CKVVESKRTR-pT-ITSQDKPS). Antibodies to TRF1^10^, TRF2^25^ and hRap1^26^ were gifts from Titia de Lange, Rockefeller University. Other antibodies used were anti-Myc (9E10, Calbiochem), anti-PML (Santa Cruz) and anti-γ-tubulin (GTU88, Sigma).

### Cell culture and retroviral infection

Cells were grown in DMEM medium with 10% fetal bovine serum (FBS) for HeLaII^16^, GM847 (a gift from Titia de Lange, Rockefeller University) and Phoenix cells^27^, supplemented with non-essential amino acids, L-glutamine, 100 U/ml penicillin and 0.1 mg/ml streptomycin. Cell lines were tested to be free of mycoplasma contamination. Retroviral gene delivery was carried out as described^18^,^19^ to generate stable cell lines. HeLaII and GM847 cells expressing pRS/pWZL, shTRF1/pWZL, shTRF1/TRF1, shTRF1/T271A or shTRF1/T271D were maintained in the selection media containing either puromycin (2 μg/ml) or hygromycin (90 μg/ml) alternating every 2 weeks for the entirety of the experiments.

### Chromatin immunoprecipitation

Chromatin immunoprecipitation (ChIP) was carried out essentially as described^16^,^28^. Each ChIP was done with 200 μl of cell lysate (equivalent to 2×10^6^ cells). Two aliquots of 50 μl supernatant (corresponding to one-quarter of the amount of lysate used for IP) were used for quantifying the total telomeric DNA and were processed along with the IP samples at the step of reversing the crosslinks. Four-fifth of immunoprecipitated DNA was loaded on the dot blots whereas two inputs each containing 5% of total DNA were included to access the consistency of loading. The ratio of the signal from each ChIP relative to the average signal from the two input lanes was multiplied by 5% (5% represents 5% of total DNA) and a factor of 1.25 (because four-fifths of the precipitated DNA was loaded for each ChIP), giving rise to the percentage of total telomeric DNA recovered from each ChIP. Detection of telomeric DNA was done with a radioactively-labelled 800-bp TTAGGG repeat-containgin fragment.

### Immunofluorescence and APB scoring

Immunofluorescence was performed essentially as described^19^. Cells were fixed at room temperature (RT) for 10 min in PBS-buffered 3% paraformaldehyde, followed by permeablization at RT for 10 min in PBS-buffered 0.5% Triton-100. Fixed cells were blocked with 0.5% bovine serum albumin (Sigma) and 0.2% gelatin (Sigma) in PBS and then incubated overnight at 4 °C with mouse anti-PML antibody in conjunction with either rabbit anti-TRF2 or rabbit anti-hRap1 antibody. Following a wash in PBS, cells were incubated with both fluorescein isothiocyanate (FITC)-conjugated donkey anti-rabbit and teramethylrhodamine isocyanate (TRITC)-conjugated donkey anti-mouse antibodies (1:250 dilution; Jackson Laboratories) at RT for 45 min. DNA was stained with 4,6-diamidino-2-phenylindole (DAPI) (0.2 μg/ml). Cell images were then recorded on a Zeiss Axioplan 2 microscope with a Hammamatsu C4742–95 camera and processed using the Openlab software package.

Scoring of APBs was done as described^29^. Cells were scored as APB positive if they contained one or more large PML bodies colocolized with TRF2 or hRap1.

### Telomere length analysis

Telomere length analysis was carried out essentially as described^12^,^30^. Briefly, genomic DNA isolated from cells was digested with *Rsa*I and *Hinf*I and loaded onto a 0.7% agarose gel in 0.5xTBE. Following electrophoresis, the gel was dried and then denatured. Blotting for telomeric fragments was carried out in gel using a radioactively-labeled single-stranded telomeric DNA probe. The average telomeric restriction fragment length was determined by PhosphorImager analysis using ImageQuant and MS excel.

### C-circle amplication assays

C-circle amplification assays were performed essentially as described^14^,^31^. Each DNA sample (50 ng) was incubated with or without 7.5 U ϕ29 DNA polymerase (NEB) in a 20 μl reaction containing 9.25 μl of premix (0.2 mg/ml BSA, 0.1% Tween 20, 1 mM ATP, 1 mM dTTP, 1 mM dGTP, 1xϕ29 buffer) at 30 °C for 8 h. Following heat inactivation of ϕ29 at 65 °C for 20 min, samples were separated on a 0.6% agarose gel in 0.5xTris-borate-EDTA (TBE) at 1.75 V/cm for 12–16 h. Gels were dried at 50 °C and were hybridized with a ^32^P-end-labeled single strand (CCCTAA)_3_ probe under native conditions as described^32^. Gels were exposed to PhosphorImager screens and scanned using a Typhoon 9400 PhosphorImager (GE Healthcare).

### Protein extracts and immunoblotting

Protein extracts and immunoblotting were performed as described^16^,^33^.

### Statistical analysis

A Student’s two-tailed *t*-test was used to derive all *P*-values.

## Additional Information

**How to cite this article**: Ho, A. *et al*. TRF1 phosphorylation on T271 modulates telomerase-dependent telomere length maintenance as well as the formation of ALT-associated PML bodies. *Sci. Rep.* **6**, 36913; doi: 10.1038/srep36913 (2016).

**Publisher’s note**: Springer Nature remains neutral with regard to jurisdictional claims in published maps and institutional affiliations.
